# Hematologic features among anemic Cameroonian pregnant women: a cross sectional study

**DOI:** 10.11604/pamj.2015.20.241.5253

**Published:** 2015-03-13

**Authors:** Elie Nkwabong, Joseph Nelson Fomulu

**Affiliations:** 1 Department of Obstetrics & Gynecology, University Teaching Hospital/ Faculty of Medicine and Biomedical Sciences, Yaoundé, Cameroon

**Keywords:** Anemia in pregnancy, Hematological features, Cameroon

## Abstract

**Introduction:**

Iron deficiency anemia is the leading cause of anemia worldwide. It may also be the leading cause of anemia in pregnancy, although this has not yet been demonstrated in our country. The aim of the study was to describe hematologic features of Cameroonian anemic pregnant women.

**Methods:**

This cross sectional analytical study was carried out in the maternity of the Yaoundé University Teaching Hospital, Cameroon, from March 1^st^, 2011 to February 28^th^, 2013. Two hundred women with singleton pregnancies and Hb concentration at booking <10 g/dl were recruited. Main variables recorded were maternal age, parity, marital status, gestational age, Hb concentration, blood group, Hb electrophoresis, mean corpuscular volume (MCV), mean corpuscular hemoglobin concentration (MCHC), pack cell volume (PCV). Data were analyzed using SPSS 18.0. P<0.05 was considered statistically significant.

**Results:**

Figures for 110 women (55%) showed microcytosis, hypochromia was observed in 122 (61%) women and megaloblastic anemia in eight women (4%). Thrombopenia was observed in 16 women (8%) and thrombocytosis in six women (3%). Anemia was microcytic hypochromic in 110 women (55%), megaloblastic in eight women (4%), normocytic hypochromic in 12 women (6%), and normocytic normochromic in 70 women (35%).

**Conclusion:**

Hematologic features of Cameroonian anemic pregnant women showed that although iron deficiency anemia is the leading cause of anemia, megaloblastic anemia is also present in our environment. A normal hematologic feature in more than the third of women shows that the cause of anemia is not always nutritional.

## Introduction

Anemia in pregnancy (AP) is defined as hemoglobin (Hb) concentration [1–3]. Because no study found significant maternal nor fetal risk when maternal Hb was ≥ 100 g/l [4, 5], AP in our setting is defined as Hb concentration [6, 7]. There are several causes of anemia. Nutritional anemia especially iron deficiency anemia is the most encountered worldwide [8, 9]. Causes also include folic acid and Vitamin B12 deficiencies. Other causes of anemia in pregnancy are hemorrhagic, hemolytic, aplastic. It can also result from ongoing chronic diseases [10]. Physiological cause is hemodilution which may be very marked is some pregnant women. Anemia is associated with increased risks of intra uterine growth restriction (IUGR), intra uterine fetal death (IUFD), pre eclampsia, preterm delivery, stillbirth, low birth weight, anemia in newborn, maternal and neonatal infections [2, 11, 12]. Cost for blood testing for iron, ferritin, serum iron binding capacity, folic acid, vitamin B12 levels is not affordable by all women in our country. Furthermore, only few laboratories can perform these tests. The causes of anemia can be suspected through the women´ hematologic features. Microcytic hypochromic red blood cells are characteristic features of iron deficiency anemia, though not specific, especially when there is positive response to iron treatment [13]. Folic acid and vitamin B12 deficiencies give macrocytic normochromic red blood cells, macro-ovalocytes, anisocytosis, poikilocytosis and hypersegmented neutrophils [14]. Both iron and folic acid/vitamin B12 deficiencies give normocytic hypochromic anemia [15]. Having the hematologic features will help us suspect the probable cause. This study looks for the causes of anemia in Cameroonian pregnant women using hematologic features.

## Methods

This cross sectional study was carried out in the maternity of the Yaoundé University Teaching Hospital, Cameroon, during a two-year period from March 1^st^, 2011 to February 28^th^, 2013. At booking each woman with a singleton pregnancy and a Hb concentration ^3^. Anemia was microcytic if MCHC and/or PCV were low. The necessary sample size was calculated as needing at least 139 women. This study received approval from the institutional ethics committee. An informed consent form was obtained from each woman. Data were analyzed using SPSS 18.0. **Footnote:** to calculate the sample size, the following formula N=P(1-P), Z_α_=α^2^/D^2^ was used where Z_α_=1.96 corresponds to a confidence level of 0.05, D=0.05 is the degree of precision and assuming that the prevalence of anemia (Hb<10 g/dl) among pregnant women (P) might be 10% in Yaoundé.

## Results

During the study period, the socio-demographic profile and blood parameters of 200 anemic pregnant women were recorded. The maternal ages varied between 17 and 45 years with a mean of 27.9 ± 5.2. Parities varied between 0 and 5 with a mean of 1.4 ± 1.4. Regarding women´s occupation, students and housewives were more represented (Table 1). The mean gestational age at booking was 21.5 ± 7.3 weeks with a range from 6 to 34 weeks. The hemoglobin concentration varied between 3.4 and 9.9 g/dl with a mean of 8.9 ± 1.1. Eight women had severe anemia (Hb concentration Table 2), while the mean corpuscular hemoglobin concentration (MCHC) was low in 46% (Table 3). The pack cell volume (PCV) was normal in 35%, low in 61% and high in 4%. The MCV, MCHC and PCV were all normal in 70 women (35%). Microcytic hypochromic anemia was noticed in 110 women (55%), megaloblastic anemia in eight women (4%), normocytic hypochromic in 12 women (6%), and normocytic normochromic in 70 women (35%). Platelet count was normal in 89%, low in 8% and high in 3% women.

**Table 1:** Distribution of women according to occupation

Occupation	Anemic women N (%)
Student	56 (28)
Housewife	46 (23)
Public sector	37 (18.5)
Private sector	16 (08)
Informal sector	39 (19.5)
Jobless	6 (03)
Total	200 (100)

**Table 2:** Distribution of mean corpuscular volumes (MCV)

MCV	Anemic women N (%)
Low	110 (55)
Normal	82 (41)
High	8 (4)
**Total**	**200**

**Table 3:** Distribution of mean corpuscular hemoglobin concentrations (MCHC)

MCHC	Anemic women N (%)
Low	92 (46)
Normal	104 (52)
High	4 (2)
**Total**	**200**

## Discussion

Regarding occupation, students were more represented. This might be due to the fact that students may have poor nutrition due to poor income. Ouédraogo et al found that under-nutrition was the leading cause of anemia in their series [16]. The gestational age at first consultation was high (21.5 weeks). This late consultation might be due to lack of financial means to start consultation and perform laboratory investigations earlier enough. In our study, almost all blood groups were found in anemic pregnant women. The hemoglobin electrophoresis of women showed that hemoglobin AA was the most represented. This can be explained by the fact that it is the most found in our country. Microcytic hypochromic anemia was found in 55% of anemic pregnant women. Hence, iron deficiency anemia is the most frequent in our setting. Thalassemia also gives microcytic hypochromic anemia, but this is very rare in our country. Megaloblastic anemia was observed in 4% of cases meaning that folic acid and/or vitamin B12 deficiencies are also present. We were unable to differentiate between folic acid and vitamin B12 deficiencies because laboratory tests to differentiate them could not be performed. The normocytic hypochromic anemia observed in 12 women (6%) is probably due to both iron and folic acid/vitamin B12 deficiencies as this has been demonstrated by some authors [15]. In sub-Saharan countries like Cameroon, many women have several nutritional deficiencies that can lead to anemia. Therefore, drugs for treating anemia in our environment should contain iron, folic acid and vitamin B12, especially if no test has to be done. Normocytic normochromic anemia was found in 70 women (35%). It might be due to recent hemolysis or hemorrhage or to hemodilution since the mean gestational age at booking was more than 20 weeks and the Hb concentration in the early gestation could not been known.

## Conclusion

Although microcytic hypochromic anemia is the most frequent type of anemia in pregnancy in our setting, megaloblastic anemia is also present. Both are present in some women. This shows that the causes of anemia is multiple in some women in Cameroon. Therefore, prevention of not only iron, but also folic acid and vitamin B12 deficiency anemia through the whole pregnancy should be systematic in all Cameroonian women. Finally, the fact that more than the third of women had normal hematologic features shows that anemia in pregnancy is not always nutritional and should be investigated.
